# The Potential of Payment for Ecosystem Services for Crop Wild Relative Conservation

**DOI:** 10.3390/plants9101305

**Published:** 2020-10-02

**Authors:** Nicholas Tyack, Hannes Dempewolf, Colin K. Khoury

**Affiliations:** 1 Graduate Institute of International and Development Studies, 1202 Geneva, Switzerland; 2 Global Crop Diversity Trust, 53113 Bonn, Germany; Hannes.dempewolf@croptrust.org*; 3 International Center for Tropical Agriculture (CIAT), 6713 Cali, Colombia; c.khoury@cgiar.org

**Keywords:** crop wild relatives, payment for ecosystem services, payment for environmental services, agrobiodiversity conservation, climate change, agricultural adaptation

## Abstract

Crop wild relatives (CWR) have proven to be very valuable in agricultural breeding programs but remain a relatively under-utilized and under-protected resource. CWR have provided resistance to pests and diseases, abiotic stress tolerance, quality improvements and yield increases with the annual contribution of these traits to agriculture estimated at USD 115 billion globally and are considered to possess many valuable traits that have not yet been explored. The use of the genetic diversity found in CWR for breeding provides much-needed resilience to modern agricultural systems and has great potential to help sustainably increase agricultural production to feed a growing world population in the face of climate change and other stresses. A number of CWR taxa are at risk, however, necessitating coordinated local, national, regional and global efforts to preserve the genetic diversity of these plants through complementary in situ and ex situ conservation efforts. We discuss the absence of adequate institutional frameworks to incentivize CWR conservation services and propose payment for ecosystem services (PES) as an under-explored mechanism for financing these efforts. Such mechanisms could serve as a potentially powerful tool for enhancing the long-term protection of CWR.

## 1. Introduction

Crop wild relatives (CWR) are wild or weedy plants that are the progenitors or close relatives of crops including those that readily cross with cultivated taxa as well as more distantly related species within the same or related genera [1]. CWR constitute genetic resources with demonstrated value for plant breeding due to their useful traits, which are not easily found in crops due to domestication bottlenecks and the subsequent narrowing of the cultivated gene pool through breeding efforts [2]. Further, CWR are often important cultural resources being directly harvested for food, spice, medicine, ceremony or other uses [3] and provide various ecosystem services within their natural habitats.

Introgression of CWR traits has led to improvements in an ever-growing list of crops including enhanced resistance to pests and diseases, tolerance to abiotic stresses such as drought, heat and salinity, yield increases, quality improvements and other desired characteristics [1,4]. Maxted and Kell identify the transfer of useful traits from 185 CWR taxa to 29 crop species [5]. Estimates of the value of these contributions have ranged from USD 8 million (USD 17.6 million in 2020) per year for increased sugar content and improved taste from a single wild tomato species [6] to USD 267–384 million (USD 310–445 million in 2020) per year for the wild sunflower gene pool [7]. Pimentel et al. estimate the contributions of CWR genetic material to increased crop yields at approximately USD 20 billion per year (USD 32.4 billion in 2020) in the United States and USD 115 billion (USD 186.3 billion in 2020) per year worldwide [8].

However, with more than a fifth of plant species worldwide threatened with extinction [9] amidst what has been called the “sixth great extinction event in Earth’s history” [10], CWR are facing threats from development and other forms of habitat modification, the industrialization of agriculture, invasive species, pollution, overharvesting, overgrazing and climate change. A recent European Red List assessment of Vascular Plants found that 11.5% of assessed CWR species in Europe were threatened with another 4.5% at Near Threatened status [11]. Jarvis et al. estimated that 16–22% of peanut, potato and cowpea wild relative species are likely to become extinct by 2055 [12] while Ureta et al. predicted that climate change is likely to lead to severe reductions in the potential distributions of maize wild relatives [13].

Up to double the amount of food that is presently produced may be required by 2050 to feed a global population of around 9 billion [14]. At the same time, normal growing season temperatures are expected to exceed the most extreme seasonal temperatures recorded from 1900 to 2006 by the end of the 21st century, which will likely have severe effects on the cultivation of many crops [15,16]. For example, rice grain yield can decline by up to 10% for each 1 °C increase in growing season minimum temperature in the dry season [17]. With rapidly evolving techniques in molecular biology, it is increasingly feasible to access and utilize genetic material from CWR including distant relatives and the expanded use of CWR with these tools provides a potential pathway to contribute to meeting these challenges through the development of crop varieties that are more resilient and productive [1,2,18]. For example, a gene from the Asian wild rice species *Oryza rufipogon* Griff. has been shown to significantly increase rice yields [19]. The incorporation of CWR derived genetic diversity into elite gene pools has been a key tool for plant breeders for many decades with hundreds of different taxa that have been used in this way especially to introduce disease tolerance traits into domesticated crops. In more recent years the value of crop wild relatives to also address abiotic stress tolerances, including many of relevance to climate change, is becoming more widely recognized and many breeding programs around the world are using these genetic resources in pursuit of that goal. As such, the range of stakeholders involved is also broadening beyond the public breeding programs in universities and national and international agricultural research programs. Several private companies are adding pre-breeders to their staff who often engage in pre-competitive public-private partnerships to utilize CWR and other types of genetic resources [2].

However, the users of CWR diversity for the most part remain far removed from where CWR are found in nature and CWR remain a largely unrecognized group within the field of conservation policy and the ecosystem services literature [20]. The continued lack of sufficient investment in CWR conservation may lead to permanent gaps in the pool of wild genetic resources available to crop breeders. Their extirpation will also have negative impacts locally including the loss of their contributions as wild-harvested plants and the disappearance of the ecosystem services they provide.

At the root of these conservation deficiencies is a lack of, or inadequacies in, institutions and payment systems by which the beneficiaries of CWR conservation services could compensate those who can supply them. Adaptation of payment for ecosystem services (PES) mechanisms to CWR offers a potentially useful tool for correcting this failure and enabling the creation of a missing institutional framework for the conservation of wild and weedy genetic diversity to support future agricultural research and crop improvement efforts. While early PES schemes have encountered challenges in implementation, the mechanism has been shown to be effective in strengthening ecosystem service provision [21] and well-designed PES programs can offer a low-cost and efficient solution for the mitigation of market failures associated with ecosystem service provision such as those associated with the carbon fixation services provided by forests or water filtration services provided by wetlands and riparian buffers [22]. The use of PES over the past years has provided potential PES practitioners with a number of lessons that can help improve program design [23]. Initial research investigating the potential for implementing PES for CWR in fact already exists in the case of several CWR taxa in Zambia where competitive tenders have been held for farmer conservation of CWR in field margins [24] as well as experiences from Latin America, which provide insights for the design of payment for agrobiodiversity conservation service programs more broadly [25]. Importantly, the successful conservation of CWR requires a combination of action on different geographic (local, national, global) and social (individual, market, societal) levels spanning in situ, ex situ and on-farm conservation [26]. If designed well, PES instruments provide the flexibility to further CWR conservation across these different dimensions.

We describe here the ways in which economic benefits flow from the conservation of CWR, discuss the absence of adequate institutional frameworks to incentivize CWR conservation services and discuss the potential of payment for ecosystem services (PES) as a tool for the conservation and sustainable use of CWR genetic diversity.

## 2. CWR and Ecosystem Services

Crop wild relative populations provide a number of ecosystem services (Table 1), which include direct contributions locally as well as cultural and genetic resource services more widely. With regard to agriculture and plant breeding, the conservation of these populations provides the important “supporting” ecosystem service of plant genetic diversity [27]. CWR germplasm, as a tangible material product resulting from ecosystem processes, is an “ecosystem good” [28] that is collected from wild, weedy or human managed habitats, deposited in gene banks or other repositories for ex situ conservation, undergoes a process of pre-breeding and breeding and finally results in the introduction of beneficial traits into crop varieties (provisioning service). In traditional farming systems, wild relatives of numerous crops also provide genetic diversity “spontaneously” to crops in the field through the natural gene flow; for example, wild and cultivated populations of cowpea often overlap and there is evidence of substantial hybridization between the two in the field [29]. The Millennium Ecosystem Assessment has also considered genetic resources as primarily a provisioning good/service [30] while other authors see genetic diversity as providing mainly a supporting service to agriculture [27]. The Economics of Ecosystems and Biodiversity (TEEB) clarifies that the collection of useful genetic resources from nature implies a provisioning service whereas the maintenance of genetic diversity, e.g., through the in situ conservation of CWR, should be considered to be a supporting or “habitat” service [31].

When CWR and other forms of agrobiodiversity are conserved and used for crop improvement, agricultural system resilience may be increased through a greater resistance to pests and diseases among other factors. Changing conditions and the outbreak of new pests and diseases can cause significant losses in productivity as occurred during the 1970–1971 Southern corn leaf blight outbreak, which led to the loss of almost 710 million bushels of the US maize crop [34]. Such epidemics can turn into catastrophes such as the Irish Potato Famine, an outbreak of the late blight disease associated with the death or displacement of 25% of the Irish population [35].

The use of CWR to breed more resistant or resilient crop varieties can help to avert persistent crop failures stemming from a genetic deficiency; for example, one of the very few potato cultivars immune to the most recent and virulent strains of late blight is Sarpo Mira with its durable, broad-spectrum resistance coming from genes that originated in the wild potato species *Solanum demissum* Lindl. [36]. The use of CWR can provide insurance value to agriculture by minimizing the risks posed by climate change, droughts and pests and diseases to the genetically homogenous monocultures of modern industrial agriculture. Though valuable traits can also be found in traditional crop varieties and other sources, in some cases the unique traits found in the genomes of CWR are essentially a non-substitutable good as shown also by the provision of cytoplasmic male sterility from sunflower CWR [37].

CWR germplasm is a mostly renewable and non-rival resource in that it can be multiplied and shared at a relatively low cost (for most crops) though it is possible to exclude others from using it. As with other forms of plant genetic resources, CWR thus constitute an imperfect public good with both public and private characteristics. Importantly, various publicly-held collections of CWR of a list of globally important crops are required to be shared freely by the International Treaty on Plant Genetic Resources for Food and Agriculture (ITPGRFA) [38], which came into force in 2004 and strengthened the public and non-excludable aspects of these resources. The ITPGRFA is a major international agreement that governs access to the CWR species of many agricultural, horticultural and forage crops. Parties are governed by the Treaty’s Multilateral System of access and benefit sharing, which specifies that publicly-held CWR materials included in Annex 1 of the ITPGRFA in countries that have ratified the Treaty are non-excludable. They must be made available to those who request them for the purpose of research and breeding, rendering these species a global public good whose benefits can be enjoyed by all although payment is required to the Treaty’s Benefit Sharing Fund if the accessed materials are used to breed a commercialized variety that itself is available without restrictions for further research and breeding. Under this Multilateral System, plant breeders and other researchers around the world have access to CWR germplasm held in the gene banks of the CGIAR and other international collections as well as the national gene banks of the countries that have ratified and implemented the Treaty (though a number of countries possessing significant CWR resources have not yet done so including China, Israel and Mexico). Non-public collections can also be placed under the umbrella of the Treaty. CWR not under the purview of the Multilateral System of the ITPGRFA fall under the governance of the Nagoya Protocol on Access and Benefit Sharing under the Convention on Biological Diversity [39] and are subject to bilateral agreement negotiations.

Land containing CWR populations maintains two important ecosystem services related to the production of the ecosystem good of CWR germplasm. First, the CWR habitat essentially serves as a natural repository for these resources. Second, it also serves as an incubator for the creation of novel allelic variation and genotypes, some of which may be useful to breeders. When the CWR habitat is destroyed, both of these services are lost as well. CWR populations often contain higher allelic diversity than cultivated diversity as a result of genetic bottlenecks and continued evolution, as has been demonstrated for soybean, and a range of CWR populations maintained across diverse topographies with different microclimates can provide both a valuable resource of genetic diversity and also incubate new traits and alleles of value [40]. Conversely, changes in the CWR habitat can destroy these resources.

In situ genetic reserves are thus an important conservation strategy, complementary to gene banks and other ex situ repositories (and vice versa) and systems employing both in situ and ex situ conservation are generally considered to be the most robust in terms of successful long-term conservation [41,42]. A population of CWR conserved in situ typically contains more genetic diversity than a single accession that is collected and stored in a gene bank, which is a subset meant to represent that population and provides a storage service associated with the conservation of genetic diversity. The incubator service refers to the maintenance of evolutionary relationships and processes in populations preserved in situ. Continued evolution of CWR populations has value for adapting crops to changes in pests and disease pressures [43]. As ecosystems change, for example through climatic shift and the subsequent migration of species, populations of CWR may adapt and these adaptations may benefit future breeding efforts. As an example, CWR living in dry ecosystems that become even drier due to climate change may respond to selection for increased drought tolerance and become particularly useful in the context of crop improvement for this trait even on short time scales [44]. It is important to note that CWR occur not only in wild ecosystems but also in disturbed and in human managed environments including in and around agricultural fields. While these may be considered as locally useful resources by farmers, wild-harvesters and others, they also may be regarded as agricultural nuisances, for example in wild pumpkins (*Cucurbita* L.), where populations close to agricultural fields in the United States and Mexico may be purposely extirpated because the gene flow can introduce bitterness into the fruit of cucurbit crops [45].

*Ex situ* conservation provides the opportunity to conserve a great diversity of samples in a small area and is thus indispensable for maintaining CWR genetic resources and facilitating their use for crop improvement. However, each sample in a gene bank only represents a single snapshot in time of a limited amount of the genetic diversity of the wild population. The in situ conservation of a CWR population coupled with ex situ protection thus provides the range of conditions under which wild populations may continue to evolve and also be readily available for agricultural research.

## 3. The Rationale for Payment for Ecosystem Services for CWR Conservation

Crop wild relatives have demonstrated economic and cultural value [46] and potentially even greater future value but major gaps have been recognized in their conservation, both in situ and ex situ. However, in spite of this demonstrated economic value and the threatened status of many CWR species, with over 70% of CWR taxa identified as high priority for further collecting [47], strong institutional frameworks to support the conservation of CWR do not exist. In the absence of such a framework, the owners of land serving as a habitat for CWR populations are not compensated for conserving CWR and thus have no incentive to do so. In fact, they may very well have various incentives to convert CWR habitat into farmland or develop it for other purposes. Indeed, it is not private actors (individuals or firms) who have implemented most of the past CWR conservation projects but rather the financing for most past major initiatives to conserve CWR in situ to date have typically come from internationally funded projects (e.g., by the Global Environmental Facility).

A key issue associated with the protection of CWR populations in situ is that many of the agricultural benefits arising from CWR conservation are separated both temporally and geographically from those who have the ability to provide them. For example, CWR might remain in a gene bank for several years or even decades before it is selected for breeding, after which it typically takes ten years for the effort to result in a released variety that provides economic benefits. 

In this paper, we explore payment for ecosystem service (PES) instruments as incentive mechanisms to provide compensation for the provision of CWR conservation services. Such market instruments have the potential to help bridge the gap between those willing to pay for CWR conservation and those able to conserve them and create stronger institutional frameworks for CWR conservation.

## 4. Payment for Ecosystem Services Mechanisms for CWR Conservation 

The adoption of payment for ecosystem services (PES) to the CWR context could incentivize the provision of CWR conservation services, bridging the gap between the beneficiaries and providers of these services. The core idea of PES is that “external (ecosystem service) beneficiaries make direct, contractual and conditional payments to local landholders and users in return for adopting practices that secure ecosystem conservation and restoration” [48].

Although payment for ecosystem services has been hailed as arguably “the most promising innovation in conservation since [the enactment of the Convention of Biological Diversity at] Rio 1992” [48], the mechanism has not been widely applied to agrobiodiversity conservation, particularly wild agrobiodiversity [49]. Noting the lack of research in this area, Narloch et al. [49] also proposed “payments for agrobiodiversity conservation services” (PACS) as a PES-like solution to the loss of landraces and other local crop varieties, which they define as an “economic instrument to tackle market, intervention and global appropriation failures associated with the public good characteristics of agrobiodiversity conservation services through the use of (monetary or in kind) reward mechanisms in order to increase the private benefits from local (plant and animal genetic resources)”.

Though PACS is designed to incentivize the on-farm conservation of under-utilized and endangered crop varieties, PES instruments are also promising in the context of mitigating the market failure associated with the under-conservation of CWR by increasing the private benefits of conserving CWR and could fund both in situ and ex situ conservation activities.

### 4.1. Who Will Pay for CWR Conservation?

Large scale CWR conservation efforts are slowly increasing at an international and various national levels as seen in the examples of the Sierra de Manantlan Biosphere reserve in Mexico, the Ammiad Project in Israel, the Erebuni Reserve in Armenia and the Global Environment Facility project “In situ conservation of crop wild relatives through enhanced information management and field application”, which developed national CWR conservation strategies in Armenia, Bolivia, Madagascar, Sri Lanka and Uzbekistan [7]. Given the value of CWR for adapting agriculture to climate change, the focus of a major CWR-related project over the past decade [50], PES mechanisms for a complementary system of in situ and ex situ conservation of CWR species may also be attractive investment targets for the proposed USD 100 billion Green Climate Fund, which envisions significant investment in climate change adaptation and mitigation measures [51].

There may also be under-explored demand for CWR conservation in the private sector. For example, the drug company Merck & Co. paid an upfront fee of USD 1 million to Costa Rica’s National Institute of Biodiversity to help conserve rainforest biodiversity in exchange for the rights to use samples of the plants, insects and microorganisms collected through this program to create new pharmaceutical products [52]. Investing in payments for CWR conservation services could provide agricultural sector companies with germplasm that might be difficult to access otherwise. A PES for CWR conservation program funded by a private agricultural firm could be seen as an investment in the long-term sustainability of the industry and could also be an opportunity for green marketing and corporate social responsibility programs [53,54]. Given that firms are under increasing pressure from stakeholders to reduce their impacts on ecosystems and biodiversity [55], companies that invest in PES for CWR conservation could gain a competitive advantage by advertising their activities through sustainability labelling programs.

Individual governments, companies or organizations may fund PES programs for CWR conservation unilaterally or could contribute to a CWR conservation fund that aims to conserve the wild genetic resources of a given crop gene pool or set of crops, providing streamlined access to contributors. The creation of CWR endowments could also aid in the sustainability and permanence of such programs. The interest from such funds would be used to pay those safeguarding the plants on a frequent basis contingent on the persistence of the CWR populations or for the maintenance of CWR within a protected area. Such programs could be arranged through already existing access and benefit sharing agreements, either bilateral (Nagoya Protocol) or multilateral (ITPGRFA) and could contribute new resources to existing funds, such as the ITPGRFA’s Benefit Sharing Fund.

A key challenge facing PES for CWR programs is that the benefits of CWR conservation are typically distant both spatially and temporally from the conservation activities (with the exception of farmers who manage CWR on-farm). In addition, given that CWR and plant genetic resources held as part of the Multilateral System as a whole act as an important global public good, the benefits of these conservation activities are highly diffused. Thus, it is likely that the majority of funds made available for CWR conservation through PES would come either from international institutions (such as the Benefit Sharing Fund of the ITPGRFA, GEF or the Green Climate Fund) or from national governments.

### 4.2. Who Will Provide CWR Conservation Services?

Crop wild relative conservation differs from the protection of cultivated crop diversity in that CWR taxa are wild species that generally do not require farmers for their persistence. That said, many CWR taxa can be weedy and can benefit from disturbance caused by humans; for example, on roadsides or on the sides of agricultural fields including CWR of maize as managed within the Sierra de Manantlan Biosphere Reserve. These plant taxa thus constitute a particular class of wild and weedy biodiversity that calls for specific forms of conservation.

While PES for CWR conservation will in some way require different designs from past PES programs, many similarities exist and lessons can be taken from past experiences with PES and PACS more specifically. Depending on the taxa, PES for in situ or on-farm CWR conservation might target farmers (as in Zambia for wild millet, cowpea and sorghum taxa [24]), private landowners, forest managers or conservationists working in the context of protected areas. In some circumstances, successful CWR conservation will require communities to collaborate who do not typically work together (e.g., conservationists/ecologists and agricultural scientists and crop breeders) to further collaborate and PES offers a tool for bridging such gaps similar to the REDD+ (Reducing Emissions from Deforestation and Forest Degradation in Developing Countries) program, which has brought together climate scientists and those working in forestry management and biodiversity conservation.

### 4.3. Towards Designing a CWR PES Conservation Portfolio

In this section, we describe a portfolio of CWR conservation actions that fit into the PES framework. These programs could also be coupled with already existing PES schemes by “bundling” CWR with other ecosystem services such as carbon fixation (e.g., REDD+) or water purification [56]. Though this portfolio focuses on in situ conservation, gene banks and other ex situ conservation repository actors are important to long-term CWR conservation and accessibility for use; thus, PES programs for in situ CWR conservation would best include integrated aspects with the ex situ conservation community. A PES scheme for CWR conservation could take many forms including:

Within preserves. CWR can be conserved through the creation of new preserves, the addition of new land containing CWR populations to existing reserves or by providing new funding to the budgets of existing reserves to support CWR conservation programs.

On farms or other highly human managed environments. Property owners could be compensated for protecting CWR populations on their lands. One manner by which to implement such work could be through the inclusion of CWR within agri-environmental payment schemes or other, already existing, PES programs such as REDD+. Many countries have agri-environmental payment programs designed to preserve the provision of environmental public goods such as biodiversity, cultural heritage and scenery. These programs theoretically have the potential to correct market failures associated with these goods [57]. Such programs could, as an example, provide compensation to farmers for conserving CWR in field margins [24] or for setting aside larger portions of their cropland as non-agricultural conservation lands. Owners and managers of roadsides could also be compensated for the protection of CWR within their mowing and herbicide use activities.

Use in landscaping, forage programs and plantations. PES could be used to fund the use of CWR in landscaping, forage programs and plantations of edible and medicinal species. These three strategies could be particularly useful for expanding the distributions of threatened CWR and may enable long-term conservation without further funding if the target species becomes sufficiently popular.

Use in restoration projects. CWR could be prioritized or subsidized for use in restoration activities in their native ranges through PES funding mechanisms.

The creation of new protected areas for CWR conservation may emphasize those that contain several important CWR populations following an optimal reserve design [58,59] as exemplified by the Sierra de Manantlan Biosphere Reserve, with a core area preserving the CWR habitat surrounded by transition and buffer zone containing settlements. Sometimes sites need not be very large, as pioneered by the plant micro-reserve initiative in Valencia, Spain [60] and the Ammiad Project in Israel, a one-hectare site that nonetheless has thus far been successful in conserving an important population of wild wheat [31]. Creating new preserves may be expensive but is sometimes necessary and feasible in areas where the presence of CWR populations overlap with high levels of other ecosystem services and species that are targeted for conservation. 

Contracting with farmers and other local people, on the other hand, could help decrease the costs of conserving a CWR population by eliminating the need to purchase the land and turn it into a protected area, allow the transfer of the payments if the CWR population shifts due to climate change and enable those administering the fund to shift the payments to other populations if the original goes extinct. For example, research conducted in Zambia to determine how much farmers would have to be paid to conserve CWR in field margins estimated conservation costs at between USD 23–91 per hectare per year [24]. Payment could be monetary or in kind. CWR conservation may also be compatible with agroforestry activities such as shade-grown coffee or cacao, allowing local people to continue economic activities in the area and farmers that take part in these programs may have the opportunity to command a premium for their products through eco-labelling, gaining multiple benefits from their involvement. As CWR are present in a wide range of habitats, this type of PES scheme for in situ conservation of CWR could also include the management of roadside CWR populations, plans for sustainable management plans of harvested populations of edible CWR and the conservation of CWR populations present in agricultural landscapes by farmers. In any such strategy, payments should be contingent on the quality of the conservation effort (generally, whether the population continues to persist, whether it increases or decreases in size, etc.) to incentivize the effective conservation of the CWR populations. Although it may prove cheaper than the creation of new CWR preserves, this strategy may be difficult to successfully design and implement due to complications with land tenure, questions about who to pay, payment structure and local culture. The long-term sustainability of the strategy will most likely be contingent upon whether or not payments continue.

### 4.4. Prioritizing CWR for Conservation

Given limited budgets, it is important to prioritize the most important resources to conserve. For CWR, the potential PES practitioner must prioritize between crop gene pools, individual species and intraspecific populations. Crop gene pools may be prioritized, among other factors, by the economic value of the crop to which they are related, the value of the crop for food security or other cultural values and/or contributions made by the crop to development and poverty alleviation. Another tool that can be used for prioritization is the so-called Weitzman approach. The Weitzman theorem uses diversity, risk status and conservation cost indices to construct a priority portfolio of conservation targets to maximize the diversity that can be conserved with any given quantity of funding [61,62]. The geographic prioritization of CWR for conservation is possible as their native distributions are known to be concentrated in primary regions of diversity around the world. Previous efforts to map the ranges of over 1000 CWR related to 81 globally important crops distinguished areas of the Mediterranean, Near East and southern Europe, South America, Southeast and East Asia and Mesoamerica as particularly CWR rich, with up to 84 taxa potentially overlapping in a 25 km^2^ area in Turkey, one of the top global hotspots for CWR diversity (an online tool has been developed that allows conservation practitioners to identify where CWR taxon richness is the highest [63]). A related gap analysis methodology has combined conservation and native distribution data on CWR to map under-collected (ex situ) and under-protected (in situ) areas, identifying priority populations for conservation [64,65].

Another important consideration is the potential usefulness of CWR species and populations to crop improvement efforts, which has been described for the CWR of many crops [66]. Consulting with breeders of the crop of interest to further determine which CWR species and populations they are most interested in may provide further context for selecting conservation targets. A maximum diversity approach might alternatively be adopted in which CWR are selected based on genetic diversity and genetic uniqueness since specific trait values are often challenging to measure in CWR [2].

## 5. Assessing the Effectiveness of PES for CWR

The success of PES mechanisms designed to conserve CWR-related ecosystem services may be assessed according to three main criteria: ecological effectiveness, economic efficiency and social equity [47,67]. This section discusses strategies for maximizing these aspects of PES programs for CWR conservation.

### 5.1. Ecological Effectiveness

The ecological effectiveness of PES schemes for CWR conservation refers to the efficacy and sustainability of programs in contributing to the preservation of the targeted CWR genetic diversity in the medium-term. A major concern of any PES scheme for the in situ conservation of CWR is how effective its payments are in preserving the ecological functions of CWR habitats and the evolutionary potential of the CWR populations. Practitioners designing a PES program for CWR conservation should assess whether or not the CWR population itself can be expected to survive indefinitely into the future; i.e., what is the risk that the population will go extinct, eliminating the benefits the payments for ecosystem services were meant to achieve. If the population is likely to go extinct in the medium-term even with conservation action, it may be a priority for collecting for ex situ conservation but may not be a wise choice for a PES program for in situ CWR protection. Methods from plant conservation biology can be utilized to set and measure the progress of populations, while studies have also predicted the effects of climate change on the range of specific CWR species into the future using methodologies that could be adapted to determine which potential preserve areas are more likely than others to remain as a suitable habitat for CWR species in future climates [10,13,68]. Last, the management of in situ CWR populations conserved through the PES mechanism should include measures to reduce the risks posed by development, livestock, crop introgression and other threats. Genetic erosion may also occur through the selection for desired traits in CWR that are used in landscaping, plantations and in forage programs and care should be taken to prevent the excessive narrowing of the conserved genetic diversity.

### 5.2. Economic Efficiency

The term economic efficiency in the context of PES for CWR conservation refers to the use of project resources such that the net economic benefit resulting from the PES program is maximized. Economic efficiency on a larger scale in this context would imply that the limited funds available for CWR conservation globally are spent in ways that maximize the economic benefits flowing from these projects (through crop improvement and other uses). Though it is difficult to predict which CWR populations will end up being most useful, the prioritization techniques discussed in Section 4.4 may indicate means by which to maximize the value of the genetic material conserved while the cost effectiveness of PES schemes for CWR conservation can be enhanced by preserving the most unique populations and/or by preserving overlapping populations of many taxa of interest. In the case of multiple habitats containing similar amounts of CWR genetic diversity, costs can be cut by selecting the habitat that is the cheapest to conserve. This holds true for the other elements of the PES programs as well, as long as the quality of the service provided remains as high as with less expensive providers. Employing a conservation auction in which potential service providers reveal their cost structures through the process of bidding may be useful in driving down the costs of conservation by helping to select the lowest-cost project partners [49]. Furthermore, it should be noted that complementary funding for the characterization and evaluation of CWR germplasm and its use in pre-breeding activities is essential for increasing the economic benefits flowing from CWR conservation.

### 5.3. Social Equity

Finally, social equity should be a key consideration for the design of PES schemes for CWR conservation. Though many authors have emphasized economic efficiency as the primary goal of PES schemes, incorporating social equity concerns is important to the success of PES mechanisms designed for CWR conservation so as to avoid the so-called “PES curse” of negative social impacts [47,69]. There may be more overlap between social equity concerns and the ecological effectiveness and economic efficiency aspects of PES than previously considered, at least for CWR.

The history of CWR conservation contains several examples of conservation programs that were designed to be both ecologically effective and socially equitable. In addition, it should be noted that even if CWR are not cultivated species, on-farm conservation techniques still are essential for the successful conservation of many CWR taxa, as shown by recent research conducted by Fagandini Ruiz et al. [70] on quinoa wild relatives in southern Peru around Lake Titicaca. Six quinoa CWR were found to be present both on permanent native meadows and cultivated land with fallow cycles and plot borders [70]. Other examples of how farmers and local communities have been included in CWR conservation efforts are numerous. For example, the Potato Park in Peru, or Parque de la Papa, is a biocultural heritage area that preserves wild relatives and landrace varieties of potato as well as other Andean crops like quinoa and oca. The park is maintained by six local Quechua communities and its management uses customary laws and institutions to aid in the conservation and sustainable use of the area’s natural resources [71]. The Global Environment Facility’s CWR Project developed a management plan for wild yams in Madagascar to allow the sustainable harvest and management for these CWR instead of simply cutting off access to the plants that locals had harvested, eaten and sold for centuries. The Sierra de Manantlan Biosphere Reserve in Mexico, with its focus on people as an integral part of the ecosystem, combines the in situ conservation of maize wild relatives and landraces with the development of local agrarian communities, ecotourism and sustainable forest management. The disease-resistant maize wild relative *Zea diploperennis* (Iltis et al.) is preserved within the core zones of the preserve with strict protection, along with *Zea perennis* (Hitchc. Reeves and Mangels) and subspecies *Zea mays* spp. *parviglumis* (H.H. Iltis and Doebley), yet around 40,000 people live in the buffer zone [72]. A project funded by the ITPGRFA’s Benefit Sharing Fund is helping to train local farmers and their families in the conservation of a maize wild relative in Nicaragua’s Apacunca Genetic Reserve and the area surrounding it as part of a wider package of development activities, seeking to involve communities in the recovery, conservation and use of teosinte (a maize CWR) while ensuring that they receive some benefit as well. Thus, local communities have played a central role in many past CWR conservation projects. 

Projects inclusive of social equity considerations tend to be internally originated and driven, owned by the community, fully backed by local practice and culture and strongly supported by other stakeholders [7]. Those designing PES schemes for CWR conservation should recognize the synergies between social equity and the ecological effectiveness and economic efficiency of CWR preservation mechanisms while at the same time acknowledging the potential tradeoffs between these goals. It might not always be possible to use these strategies but social equity considerations should at least be considered during PES mechanism design. Participatory approaches present opportunities for CWR conservation to bring added benefits through the social and economic empowerment of often-marginalized groups by sharing the benefits of the program with those who live nearby; for example, through their involvement in the planting of CWR species or in the maintenance of plantations of edible CWR and in training and job creation in ecotourism activities centered on CWR. They may also help to tap into local ethnobotanical knowledge through the engagement of local parabotanists who may be better suited to identify, manage and educate others about the CWR resources of a particular area. For example, local rural communities were found to have a detailed knowledge of the utility of the flora in the Sierra de Manantlan, using more than 500 of the plant species present in the area [73].

## 6. Conclusions

Payment for ecosystem services (PES) has been shown to be an effective mechanism mitigating market failures associated with the provision of ecosystem services such as water filtration, carbon fixation and a number of other economically and culturally valuable functions provided by the natural world. In this article, we argue that PES may offer a useful tool for ensuring the conservation of priority, at-risk populations of CWR of important crops and may assist CWR conservation efforts at a local, national, regional and global scale.

Currently, adequate institutional frameworks to support the conservation of CWR worldwide do not exist, in part due to insufficient incentives for providing CWR conservation services. Payment for ecosystem services could be a promising tool for solving this problem by directly linking payments from public and private beneficiaries of CWR conservation services to their suppliers, bridging substantial spatial and temporal gaps. PES in particular offers a flexible mechanism for advancing CWR conservation that may prove successful in a broad range of situations and scenarios in developing and developed countries alike. 

The loss of CWR genetic diversity results in the irreversible destruction of resources of significant importance to the sustainability and resilience of future agriculture, to local food and cultural security and to the provision of local ecosystem services. To ensure that this diversity is present and available when needed, it is necessary that investments be made in the conservation of CWR. The payment for ecosystem services (PES) mechanism has the potential to aid in this goal by providing economic incentives for the maintenance of CWR resources.

## Figures and Tables

**Table 1:** List of ecosystem services provided by CWR.

Ecosystem Service	Examples
Supporting service	The conservation of CWR populations maintains genetic diversity and allows for the continuing evolution of the gene pool as a resource for future crop improvement, providing an important supporting service to help meet future demand for improved crop varieties and resilient agricultural systems [32].
Regulating services Provisioning services Cultural services	CWR can regulate certain ecosystem processes such as pest and disease control, pollination efficiency, nutrient cycling, decomposition, erosion control and carbon sequestration [27]. CWR provide a provisioning service of genetic resources when CWR germplasm is collected from wild populations and used by plant breeders to develop improved varieties. In addition, some CWR are harvested and directly used for food, spice, medicine, ceremony or other purposes [28]. CWR are a part of the world’s natural and cultural heritage with potential for ecotourism (e.g., wild coffee forests in Ethiopia) [33].
